# Serotype Replacement after Introduction of 10-Valent and 13-Valent Pneumococcal Conjugate Vaccines in 10 Countries, Europe

**DOI:** 10.3201/eid2801.210734

**Published:** 2022-01

**Authors:** Germaine Hanquet, Pavla Krizova, Tina Dalby, Shamez N. Ladhani, J. Pekka Nuorti, Kostas Danis, Jolita Mereckiene, Mirjam J. Knol, Brita A. Winje, Pilar Ciruela, Sara de Miguel, Maria Eugenia Portillo, Laura MacDonald, Eva Morfeldt, Jana Kozakova, Palle Valentiner-Branth, Norman K. Fry, Hanna Rinta-Kokko, Emmanuelle Varon, Mary Corcoran, Arie van der Ende, Didrik F. Vestrheim, Carmen Munoz-Almagro, Juan-Carlos Sanz, Jesus Castilla, Andrew Smith, Birgitta Henriques-Normark, Edoardo Colzani, Lucia Pastore-Celentano, Camelia Savulescu, the SpIDnet group

**Affiliations:** Epiconcept, Paris, France (G. Hanquet, C. Savulescu);; Antwerp University, Antwerp, Belgium (G. Hanquet);; National Institute of Public Health, Prague, Czech Republic (P. Krizova, J. Kozakova);; Statens Serum Institut, Copenhagen, Denmark (T. Dalby, P. Valentiner-Branth);; Public Health England, London, UK (S.N. Ladhani, N.K. Fry);; Tampere University, Tampere, Finland (J.P. Nuorti);; Finnish Institute for Health and Welfare, Helsinki, Finland (J.P. Nuorti, H. Rinta-Kokko);; Santé Publique France, the National Public Health Agency, Saint-Maurice, France (K. Danis);; Health Protection Surveillance Centre, Dublin, Ireland, UK (J. Mereckiene);; National Institute for Public Health and the Environment, Bilthoven, the Netherlands (M.J. Knol);; Norwegian Institute of Public Health, Oslo, Norway (B.A. Winje, D.F. Vestrheim);; Public Health Agency of Catalonia, Barcelona, Spain (P. Ciruela);; CIBER Epidemiología y Salud Pública, Madrid, Spain (P. Ciruela, C. Munoz-Almagro, J. Castilla);; General Directorate of Public Health–Madrid Region, Madrid (S. de Miguel, J.-C. Sanz);; Navarra Hospital Complex–IdiSNA, Pamplona, Spain (M.E. Portillo);; Public Health Scotland, Glasgow, Scotland, UK (L. MacDonald);; Public Health Agency of Sweden, Stockholm, Sweden (E. Morfeldt);; Centre de Recherche Clinique et Biologique, Creteil, France (E. Varon);; Children’s Health Ireland (CHI) at Temple Street, Dublin (M. Corcoran);; Academic Medical Center, Amsterdam, the Netherlands (A. van der Ende);; Hospital Sant Joan de Déu, Barcelona (C. Munoz-Almagro);; Universitat Internacional of Catalunya, Barcelona (C. Munoz-Almagro);; Public Health Institute of Navarra–IdiSNA, Pamplona (J. Castilla);; University of Glasgow, Glasgow, Scotland, UK (A. Smith);; Karolinska Institutet, Stockholm (B. Henriques-Normark); K; arolinska University Hospital, Solna, Sweden (B. Henriques-Normark);; European Centre for Disease Prevention and Control, Stockholm (E. Colzani, L. Pastore-Celentano)

**Keywords:** *Streptococcus pneumoniae*, pneumococcal infections, 13-valent pneumococcal vaccine, 10-valent pneumococcal vaccine, 15-valent pneumococcal vaccine, 20-valent pneumococcal vaccine, invasive pneumococcal disease, serotype, respiratory infections, SpIDnet Multicenter Study, bacteria

## Abstract

We evaluated invasive pneumococcal disease (IPD) during 8 years of infant pneumococcal conjugate vaccine (PCV) programs using 10-valent (PCV10) and 13-valent (PCV13) vaccines in 10 countries in Europe. IPD incidence declined during 2011–2014 but increased during 2015–2018 in all age groups. From the 7-valent PCV period to 2018, IPD incidence declined by 42% in children <5 years of age, 32% in persons 5–64 years of age, and 7% in persons >65 years of age; non-PCV13 serotype incidence increased by 111%, 63%, and 84%, respectively, for these groups. Trends were similar in countries using PCV13 or PCV10, despite different serotype distribution. In 2018, serotypes in the 15-valent and 20-valent PCVs represented one third of cases in children <5 years of age and two thirds of cases in persons >65 years of age. Non-PCV13 serotype increases reduced the overall effect of childhood PCV10/PCV13 programs on IPD. New vaccines providing broader serotype protection are needed.

Starting in 2010–2011, most countries in Europe progressively replaced the 7-valent pneumococcal conjugate vaccines (PCV7) in the infant immunization schedule with the 10-valent (PCV10) vaccine, 13-valent (PCV13) vaccine, or both (Figure 1) (*1*). Programs using these vaccines have substantially reduced the burden of invasive pneumococcal disease (IPD) caused by vaccine serotypes in children and in unvaccinated adults through indirect (herd) protection (*2*–*4*). However, concomitant year-to-year increases of IPD incidence caused by nonvaccine serotypes have also been reported in children and adults in most European Union (EU) countries, which have restricted the overall benefits of the infant program on the overall IPD incidence (*3*–*6*). These rises are primarily attributable to vaccine introduction; however, other factors, such as natural trends in IPD caused by individual serotypes, might also play a role (*7*–*10*). In addition, in some countries, such as the United States, no increase in non-PCV13 serotype disease after PCV13 implementation in 2010 has been observed (*11*). Several hypotheses have been proposed to explain such differences, but none have been verified (*8*,*12*).

**Figure 1:**
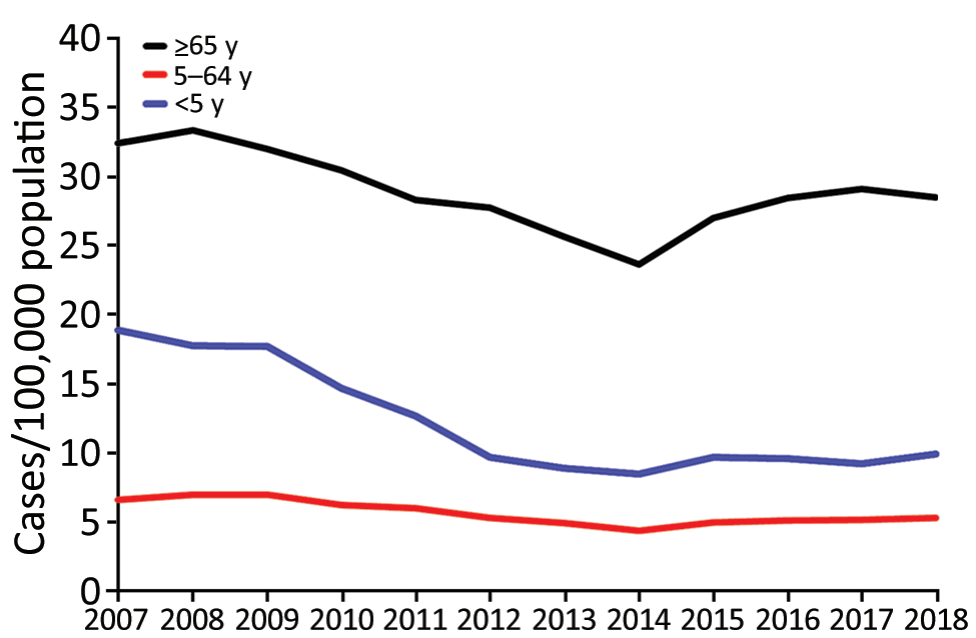
Overall incidence rates of invasive pneumococcal disease (pooled) per year, by age group in 13 SpIDnet (*Streptococcus pneumoniae* Invasive Disease network) sites, Europe.

Given the limited success of past and current PCVs on the extent of IPD in many countries, several questions need to be answered to inform policymakers. In particular, IPD burden in older adults remains very high, and whether this group should receive pneumococcal conjugate or polysaccharide vaccine is unclear. PCV13 is authorized in older adults, but the PCV13 serotype group covers a limited and decreasing proportion of IPD cases in this group because of the indirect effect of childhood PCV10/PCV13 programs on PCV13 serotype incidence (*1*,*3*). The pneumococcal polysaccharide 23-valent vaccine (PPV23) covers 12 of the PCV13 serotypes and 11 additional non-PCV13 serotypes, which were responsible for 72% of IPD cases in EU countries in 2017 across all age groups (*1*). However, its efficacy against pneumococcal disease, including pneumonia, and its duration of protection remain controversial (*13*,*14*). New higher-valent conjugate vaccines containing 15 (PCV15) and 20 (PCV20) serotypes (*15*,*16*) have been approved, but the proportion of cases these could prevent and its evolution over time is poorly documented.

SpIDnet (*Streptococcus pneumoniae* Invasive Disease network) was established in 2012, ideated and funded by the European Centre for Disease Prevention and Control to assess the effect of pneumococcal conjugate vaccination on IPD in the EU (*3*,*4*). SpIDnet collected IPD data from 13 sites across 10 countries: the Czech Republic, Denmark, England, Finland, France, Ireland, the Netherlands, Norway, Scotland, Sweden, and Spain (the Madrid, Catalonia, and Navarra regions). PCV13, PCV10, or both were introduced in these sites during 2010–2011 (Table 1).

**Table 1:** PCV program uptake and history in 13 SpIDnet sites according to national and ECDC reports, Europe, 2011–2018*

Site	Introduction of childhood PCV7	Introduction of PCV10 or PCV13 and schedule	2011	2012	2013	2014	2015	2016	2017	2018	Vaccination of persons >65 y in 2018 (uptake on all, 2015–2018)	No PCV7 y included
CZ	Not universal, recommended and used in 2009	Universal PCV10 and PCV13 in 2010 (≈equal shares), 3+1 doses	81%	80%	77%	74%	71%	68%	67%	64%	PCV13 (low) + PPV23	1
DK	2007, universal	Universal PCV13 in 2010, 2+1 doses	89%	90%	91%	91%	91%	94%	96%	96%	PCV13 (6%) + PPV23 (11%)	3
EN	2006, universal	Universal PCV13 in 2010, 2+1 doses	94%	94%	94%	94%	94%	94%	93%	93%	PPV23 (70%)	3
FI	Not introduced	Universal PCV10 in 2010, 2+1 doses	90%	94%	94%	95%	96%	96%	96%	94%	Groups at risk: PCV13 (12%) + PPV23 (2%)	0†
FR	2003 for children at risk, 2006 for all <2 y	Universal PCV13 in 2010, 2+1 doses	94%	94%	95%	94%	95%	96%	95%	98%	Groups at risk: PCV13 (4%) + PPV23 (7%)	6
IE	2008, universal	Universal PCV13 in 2010, 2+1 doses	90%	93%	93%	92%	93%	91%	90%	93%	PPV23 (36%), PCV13 in groups at risk	2
NL	2006, universal	Universal PCV10 in 2011, 2+1 doses	95%	95%	95%	94%	94%	94%	93%	93%	Groups at risk: PPV23 (PPV23 for all in 2020)	5
NO	2006, universal	Universal PCV13 in 2011, 2+1 doses	93%	93%	93%	93%	93%	94%	92%	93%	PPV23 (15%)	5
SC	2006, universal	Universal PCV13 in 2010, 2+1 doses	94%	95%	96%	96%	95%	95%	95%	95%	PPV23 (68%)	3
SE	2009, universal	Universal PCV10 and PCV13 in 2010 (≈equal shares), 2+1 doses	98%	98%	97%	97%	97%	97%	97%	97%	Groups at risk: PPV23	1
CAT	2001 for groups at high risk and recommended for all‡	PCV13 recommended since 2010, universal since 2016, 3+1 doses§	≈50%	≈50%	≈50%	≈50%	73%	73%	82%	93%	PPV23 (60%)	4
MAD	2006, universal	Universal PCV13 in 2010, interrupted in 2012–2014, 2+1 doses	100%	92%	77%	77%	99%	99%	92%	96%	PCV13¶ (since 2018, 9%) + PPV23 (71%)	3
NAV	2001 for groups at high risk and recommended for all‡	PCV13 recommended since 2010, universal since 2016, 3+1 doses§	70%	73%	75%	78%	81%	88%	88%	81%	PPV23 (57%)	5

*Green denotes universal PCV10 and PCV13; dark blue denotes universal PCV13; light blue denotes PCV13 recommended (not universal); orange denotes universal PCV10. CAT, Catalonia; CZ, Czech Republic; DK, Denmark; ECDC, European Centre for Disease Prevention and Control; EN, England; FI, Finland; FR, France; IE, Ireland; MAD, Madrid; NAV, Navarra; NL, the Netherlands; NOR, Norway; PCV, pneumococcal conjugate vaccine; PCV7, 7-valent pneumococcal conjugate vaccine; PCV10, 10-valent pneumococcal conjugate vaccine; PCV13, 13-valent pneumococcal conjugate vaccine; PPV23, 23-valent pneumococcal polysaccharide vaccine; SC, Scotland; SE, Sweden; SpIDnet, *Streptococcus pneumoniae* Invasive Disease network. †Because PCV7 was not used, the years 2005–2008 have been used as pre-PCV10/PCV13 years. ‡Recommended by pediatricians and not funded; uptake ≈50%. §PCV13 used almost exclusively; the PCV10 uptake in children <2 years of age was minimal (<1% in NAV and <5% in CAT). ¶PCV13 recommended in 2016 and 2017 for those 60 years of age, and since 2018 for all persons >60 years of age.

In this study, we estimate the overall effect (direct and indirect) of the childhood PCV10/PCV13 program on IPD across 13 sites in Europe during the first 8 years of the program. We also describe trends in serotypes included in current and future vaccines, by age group, to support decision-making on pneumococcal vaccination policies.

## Materials and Methods

Using surveillance data provided by SpIDnet sites, we estimated the overall effect of the childhood PCV10/PCV13 program by comparing IPD incidence before and after vaccine introduction, as described elsewhere (*3*,*4*,*17*,*18*). We describe the main features in this section.

### Data Sources

We conducted enhanced population-based IPD surveillance in the 13 sites by using a common protocol to ensure comparable approaches for data collection and analysis (*17*). Reporting of IPD cases, referral of strains, or both were mandatory in 10 sites and voluntary in 3 sites. The serotype responsible for IPD was determined at national and regional reference laboratories as described elsewhere (*3*,*4*). We collected data from all surveillance sites on laboratory-confirmed IPD cases (European Centre for Disease Prevention and Control 2012 definition) (*19*) by calendar year during PCV7 and PCV10/PCV13 periods. We classified serotypes into vaccine categories (Table 2). The study was embedded in IPD routine surveillance at individual sites and conducted according to local ethics regulations. Data reported by sites were anonymized.

**Table 2:** Categories of pneumococcal serotypes according to vaccine serotypes*

Category	Serotypes included
PCV7	4, 6B, 9V, 14, 18C, 19F, and 23F
PCV10 non-7 (in PCV10 and not PCV7)	1, 5, and 7F
PCV13 non-10 (in PCV13 and not PCV10)	3, 6A, and 19A
Non-PCV10	Any serotype not in PCV10
Non-PCV13	Any serotype not in PCV13
PPV23 non-PCV13 (in PPV23 and not PCV13)	2, 8, 9N, 10A, 11A, 12F, 15B, 17F, 20, 22F, and 33F
Nonvaccine (not in PCV13 or PPV23)	Any serotype not in PCV13 and not in PPV23
New vaccines	
PCV15 non-13 (in PCV15 and not PCV13)	22F and 33F
PCV20 non-13 (in PCV20 and not PCV13)	8, 10A, 11A, 12F, 15B, 22F and 33F

*PCV, pneumococcal conjugate vaccine; PCV7, 7-valent pneumococcal conjugate vaccine; PCV10, 10-valent pneumococcal conjugate vaccine; PCV13, 13-valent pneumococcal conjugate vaccine; PPV23, 23-valent pneumococcal polysaccharide vaccine.

### Data Analysis

For each site, we accounted for missing serotype data by assuming the same serotype distribution in cases with and without serotype information, by year and age group. For 3 sites in which a change in surveillance sensitivity over the surveillance period was reported (Czech Republic, Denmark, and France), we adjusted the number of reported cases to the sensitivity for each period, as described elsewhere (*20*,*21*).

We computed annual incidence rates by age group (<5, 5–64, and >65 years) and incidence rate ratios (IRR) per site, overall and by serotype category, and by age group. We compared the IPD incidence rate during the childhood PCV10/PCV13 program (2011–2018) to the average incidence rate during the PCV7 period, which varied across sites (Table 1), and to the years 2005–2008 for Finland, where PCV7 was not introduced. We performed sensitivity analyses by using the 2009 incidence rate.

We computed pooled IRR and 95% CI, overall and by serotype category, by using random effects meta-analysis, because we assumed that the actual indirect effect could vary across sites (*22*). In the <5 years and >65 years age groups, we calculated IRR by pooling sites with similar program characteristics in terms of PCV history and uptake (Table 1). We compared the 6 sites with a universal PCV13 program during the study period, the 3 sites in Spain (in which a universal PCV13 program was delayed or interrupted during the study period), and the 4 sites using PCV10 (with or without PCV13). We also compared the 9 sites with high vaccine uptake (>90% in all years during 2012–2018) and the 4 sites with moderate uptake (50%–89% in >1 year during 2012–2018; uptake in Madrid was 96% in 2018 but 77% in 2013–2014 because of program interruption). Finally, we compared the 9 sites with a long PCV7 immunization period (>3 years) and the 4 sites with short (<3 years) or no PCV7 immunization period, which included 3 sites using PCV10.

We assessed statistical heterogeneity by estimating the between-study variance by using 𝜏^2^ (*23*). Heterogeneity between sites was considered low if 𝜏^2^<0.2, fairly reasonable for 𝜏^2^ 0.2–0.5, and fairly high for 𝜏^2^ >0.5 and <1.0 (*2*). The PCV10/PCV13 overall effect was expressed as percentage change in incidence ([IRR–1] × 100).

We computed the percentage of each serotype group covered by the current and future PCV, per year, on the basis of serotype-specific data provided by a subset of sites (12 sites for the <5 years age group, 10 sites for the >65 years age group; Sweden was excluded in both age groups, and Finland and France were excluded for the >65 years age group). We analyzed separately serotype 3 distribution because it is unknown whether these vaccines protect against serotype 3 (*24*). Proportions were compared by using the χ^2^ test and a p value <0.05 was considered significant. Statistical analyses were performed by using Stata version 15.1 (StataCorp, https://www.stata.com).

## Results

A total of 17,302 cases were reported in 2018, including 964 in children <5 years of age, 6,928 in persons 5–64 years of age, and 9,410 in persons >65 years of age. In the <5 years age group, 81% of IPD isolates were serotyped; 90% of IPD isolates were serotyped in the 5–64 years age group; and 91% of IPD isolates were serotyped for cases in persons >65 years of age, excluding France (*20*). (In France, the incidence and serotype distribution are reported through 2 different systems without case reconciliation. In 2018, Norway had a very low serotyping rate, ranging from 40% to 47% per age group).

### Change in IPD Incidence, Pooled Analysis of All Sites

IPD incidence varied across sites, age groups, and over time (Table 3; Figure 1; Appendix Figure 1). In 2018, rates per 100,000 population were highest in persons >65 years of age (pooled incidence 28.5), lowest in persons 5–64 years of age (pooled incidence 5.3), and ranged from 4.6 to 29.1 in children <5 years of age (pooled incidence 9.9). The pooled incidence in each age group gradually declined to reach a minimum in 2014 and increased during 2015–2018 (Figure 1).

**Table 3:** Population size by age group and site in 2018 and IPD incidence rates per PCV10/PCV13 year during 2011–2018 by age group and site, SpIDnet multicenter study, Europe*

Sites	Population, 2018	Annual incidence rate							
		2011	2012	2013	2014	2015	2016	2017	2018
In children <5 y									
CZ	567,172	4.2	2.6	5.1	4.7	4.0	2.4	2.9	4.6
DK	300,798	10.1	11.6	11.2	11.7	7.5	8.1	9.6	5.7
EN	3,515,430	9.5	6.6	7.1	7.7	8.6	8.5	7.8	8.2
FI	267,686	23.8	10.5	10.9	9.0	7.8	11.5	10.8	12.8
FR	2,655,339	13.5	10.7	8.4	7.1	9.4	9.5	9.2	10.0
IE	319,296	11.5	11.8	10.7	10.2	9.7	11.8	12.6	15.0
NL	217,025	7.8	4.8	3.1	7.1	6.8	5.0	7.3	7.4
NO	294,863	9.1	6.1	8.3	6.1	6.2	8.5	4.6	6.8
SC	276,862	12.1	11.5	12.2	8.9	16.1	14.8	14.9	15.2
SE	604,498	7.1	4.7	5.7	5.8	3.9	5.7	4.7	6.8
CAT	359,195	41.7	38.0	25.2	24.5	30.3	26.4	25.7	28.4
MAD	320,026	23.3	16.6	16.1	15.8	18.7	17.2	18.7	17.6
NAV	30,958	20.1	14.3	28.7	33.7	42.6	24.9	28.6	29.1
All	9,729,148	12.6	9.7	8.9	8.5	9.7	9.6	9.2	9.9
In persons 5–64 y									
CZ	7,996,011	2.9	2.3	2.7	2.0	2.6	1.8	2.4	2.5
DK	4,364,329	9.7	8.6	9.1	5.9	6.5	6.3	6.5	6.1
EN	44,769,133	5.1	4.6	4.2	4.2	5.3	5.8	5.6	5.5
FI	4,045,396	9.9	9.2	9.1	7.9	9.0	9.1	8.7	8.3
FR	35,961,594	6.1	5.0	4.3	3.6	3.7	3.9	4.0	4.2
IE	3,864,357	4.0	3.5	3.7	3.9	3.9	4.1	4.4	5.1
NL	3,268,467	8.6	8.2	8.2	6.7	7.7	7.0	7.1	7.5
NO	4,114,508	8.2	7.2	7.3	6.5	4.8	4.8	5.3	5.2
SC	4,135,124	6.0	5.1	6.4	4.6	6.7	8.0	7.3	6.8
SE	7,589,976	8.3	7.9	6.9	5.6	6.8	6.1	6.4	6.8
CAT	5,767,319	7.3	7.5	6.7	5.9	6.4	6.0	7.1	7.2
MAD	5,066,849	4.3	3.5	3.3	3.8	4.4	4.2	5.1	5.5
NAV	489,838	4.5	5.3	3.3	5.6	7.4	4.7	4.8	5.5
All	131,432,901	6.0	5.3	4.9	4.4	5.0	5.1	5.2	5.3
In persons >65 y									
CZ	2,086,617	8.0	8.2	10.2	8.3	9.9	8.3	11.7	12.5
DK	1,116,063	57.8	56.2	47.5	45.0	51.1	44.0	44.7	49.7
EN	10,831,246	23.0	22.4	21.4	21.4	26.3	29.6	30.1	29.3
FI	1,192,077	30.3	33.6	30.2	32.5	38.0	36.1	37.2	32.9
FR	9,813,812	29.2	26.5	22.0	18.4	19.8	21.7	22.2	20.9
IE	673,362	30.1	32.2	30.1	28.6	31.2	30.1	31.9	39.4
NL	809,073	51.5	53.0	53.4	42.9	53.5	50.7	46.7	52.9
NO	897,368	51.3	42.6	38.8	38.3	37.4	44.1	37.8	38.0
SC	1,026,114	24.4	25.7	30.1	22.8	31.8	34.0	37.0	28.8
SE	2,035,711	41.6	45.5	42.1	38.8	41.1	43.7	42.6	41.7
CAT	1,417,311	27.2	32.1	34.5	29.0	33.0	31.0	34.4	37.5
MAD	1,154,255	18.9	19.1	17.9	22.6	23.5	24.5	26.6	23.9
NAV	126,482	29.2	29.5	22.2	20.6	24.7	28.5	36.1	25.3
All	33,179,491	28.3	27.7	25.6	23.6	27.0	28.5	29.1	28.5

*CAT, Catalonia; CZ, Czech Republic; DK, Denmark; EN, England; FI, Finland; FR, France; IE, Ireland; IPD, invasive pneumococcal disease; MAD, Madrid; NAV, Navarra; NL, the Netherlands; NOR, Norway; PCV, pneumococcal conjugate vaccine; SC, Scotland; SE, Sweden; SpIDnet, *Streptococcus pneumoniae* Invasive Disease network.

In the <5 years age group, an initial 49% decline in overall IPD incidence between the PCV7 period and 2014 was followed by a 17% increase during 2015–2018 compared with 2014, which resulted in a net 42% decline between the PCV7 period and 2018 (Table 4; Figure 1). Over the same period, PCV7 vaccine serotype IPD declined by 88%, PCV10 non-7 vaccine serotype IPD (serotypes in PCV10 and not PCV7) declined by 95%, and PCV13 non-10 vaccine serotype IPD declined by 46%, whereas serotype 3 fluctuated and returned to PCV7 values in 2018. Non-PCV13 IPD increased gradually until 2018, when it exceeded by 111% the PCV7 period incidence.

**Table 4:** IPD incidence per PCV10/PCV13 year during 2011–2018 compared with the PCV7 period by age group in the 13 sites of SpIDnet multicenter study, Europe*

Serotype group	Incidence rate ratio (95% CI)							
	2011	2012	2013	2014	2015	2016	2017	2018
In children <5 y								
All types	0.70 (0.6–0.79)	0.52 (0.43–0.63)	0.53 (0.45–0.62)	0.51 (0.43–0.60)	0.54 (0.44–0.66)	0.54 (0.45–0.64)	0.54 (0.44–0.65)	0.58 (0.49–0.70)
PCV7	0.22 (0.12–0.40)	0.18 (0.11–0.29)	0.13 (0.07–0.23)	0.11 (0.05–0.23)	0.11 (0.06–0.23)	0.09 (0.04–0.20)	0.10 (0.05–0.20)	0.12 (0.07–0.22)
PCV10–non-7†	0.59 (0.48–0.74)	0.36 (0.26–0.48)	0.22 (0.13–0.36)	0.13 (0.07–0.24)	0.07 (0.04–0.11)	0.05 (0.03–0.11)	0.03 (0.02–0.06)	0.05 (0.02–0.12)
Non-PCV10	1.09 (0.89–1.34)	0.91 (0.74–1.11)	1.06 (0.85–1.33)	1.08 (0.87–1.34)	1.18 (0.98–1.43)	1.21 (0.98–1.49)	1.21 (0.98–1.48)	1.34 (1.07–1.68)
PCV13–non-10‡	0.77 (0.61–0.98)	0.40 (0.27–0.61)	0.45 (0.31–0.67)	0.37 (0.23–0.58)	0.35 (0.21–0.60)	0.50 (0.32–0.78)	0.47 (0.30–0.73)	0.54 (0.32–0.93)
Serotype 3	0.97 (0.71–1.33)	0.58 (0.34–0.99)	0.80 (0.57–1.11)	0.60 (0.40–0.89)	0.64 (0.44–0.92)	1.02 (0.74–1.40)	1.01 (0.73–1.39)	1.03 (0.67–1.58)
Non-PCV13	1.39 (1.05–1.85)	1.47 (1.15–1.88)	1.63 (1.28–2.09)	1.76 (1.40–2.21)	2.03 (1.65–2.50)	1.93 (1.52–2.44)	1.93 (1.54–2.43)	2.11 (1.68–2.65)
In persons 5–64 y								
All types	0.87 (0.79–0.96)	0.78 (0.71–0.85)	0.76 (0.69–0.84)	0.66 (0.61–0.71)	0.75 (0.67–0.84)	0.72 (0.63–0.83)	0.76 (0.69–0.85)	0.78 (0.71–0.87)
PCV7	0.45 (0.32–0.62)	0.28 (0.20–0.42)	0.25 (0.17–0.37)	0.16 (0.10–0.24)	0.17 (0.11–0.24)	0.14 (0.11–0.20)	0.15 (0.10–0.23)	0.17 (0.12–0.24)
PCV10–non-7†	0.83 (0.70–1.00)	0.61 (0.49–0.76)	0.54 (0.42–0.69)	0.34 (0.27–0.44)	0.22 (0.15–0.32)	0.12 (0.08–0.18)	0.09 (0.06–0.13)	0.07 (0.04–0.11)
PCV13–non-10‡	1.10 (1.01–1.19)	0.92 (0.79–1.07)	0.95 (0.77–1.19)	0.77 (0.59–0.99)	0.90 (0.67–1.19)	0.89 (0.65–1.22)	0.99 (0.72–1.36)	1.06 (0.80–1.41)
Serotype 3§	1.03 (0.90–1.17)	1.03 (0.86–1.22)	1.10 (0.86–1.42)	0.90 (0.73–1.10)	1.03 (0.86–1.22)	1.06 (0.80–1.40)	1.19 (0.92–1.54)	1.28–1.01–1.63)
Non-PCV13	1.04 (0.91–1.19)	1.14 (1.02–1.28)	1.17 (1.05–1.31)	1.21 (1.08–1.35)	1.48 (1.28–1.71)	1.59 (1.37–1.85)	1.61 (1.41–1.84)	1.63 (1.42–1.88)
In persons >65 y								
All types	0.86 (0.81–0.92)	0.87 (0.82–0.92)	0.84 (0.78–0.90)	0.78 (0.71–0.85)	0.89 (0.79–1.00)	0.90 (0.80–1.00)	0.94 (0.83–1.07)	0.93 (0.82–1.05)
PCV7	0.37 (0.29–0.47)	0.29 (0.20–0.41)	0.24 (0.16–0.34)	0.19 (0.13–0.27)	0.17 (0.11–0.26)	0.15 (0.10–0.22)	0.17 (0.11–0.24)	0.14 (0.09–0.20)
PCV10–non-7†	0.90 (0.77–1.06)	0.81 (0.67–0.98)	0.56 (0.46–0.68)	0.44 (0.34–0.58)	0.27 (0.19–0.39)	0.16 (0.11–0.23)	0.08 (0.05–0.12)	0.09 (0.06–0.14)
PCV13–non-10‡	1.02 (0.93–1.12)	0.89 (0.79–1.00)	0.87 (0.71–1.07)	0.73 (0.58–0.92)	0.87 (0.68–1.11)	0.88 (0.71–1.10)	0.95 (0.75–1.20)	0.97 (0.73–1.29)
Serotype 3	0.99 (0.87–1.12)	0.93 (0.82–1.05)	0.99 (0.82–1.21)	0.93 (0.72–1.19)	1.11 (0.89–1.37)	1.22 (1.05–1.42)	1.28 (1.08–1.50)	1.32 (1.05–1.66)
Non-PCV13	1.20 (1.09–1.34)	1.40 (1.30–1.51)	1.44 (1.33–1.54)	1.44 (1.34–1.55)	1.74 (1.58–1.90)	1.81 (1.61–2.03)	1.88 (1.68–2.11)	1.84 (1.69–2.00)
PPV23– non-PCV13¶	1.14 (0.97–1.35)	1.25 (1.12–1.41)	1.30 (1.17–1.44)	1.30 (1.20–1.41)	1.63 (1.50–1.76)	1.75 (1.54–1.99)	1.83 (1.65–2.03)	1.76 (1.61–1.92)
Nonvaccine#	1.31 (1.11–1.54)	1.65 (1.40–1.95)	1.71 (1.43–2.05)	1.67 (1.39–2.01)	1.97 (1.65–2.36)	1.97 (1.66–2.35)	2.03 (1.63–2.54)	2.13 (1.81–2.50)

*See Table 1 for PCV7 period. IPD, invasive pneumococcal disease; PCV, pneumococcal conjugate vaccine; PCV7, 7-valent pneumococcal conjugate vaccine; PCV10, 10-valent pneumococcal conjugate vaccine; PCV13: 13-valent pneumococcal conjugate vaccine; PPV23, 23-valent pneumococcal polysaccharide vaccine; SpIDnet, *Streptococcus pneumoniae* Invasive Disease network. †Serotypes 1, 5, and 7F. ‡Serotypes 3, 6A, and 19A. §12 sites (all sites except Sweden). ¶Serotypes 2, 8, 9N, 10A, 11A, 12F, 15B, 17F, 20, 22F, and 33F. #Serotypes not in PPV23 and not in PCV13.

Among persons 5–64 years of age, an initial 34% decline in overall IPD incidence between the PCV7 period and 2014 was followed by an 18% increase during 2015–2018, resulting in a net 22% decline between the PCV7 period and 2018 (Table 4; Figure 1). Over the same period, IPD incidence caused by PCV7 and PCV7 non-10 serotypes declined by 83%–93%, whereas PCV13 non-10 serotype incidence declined until 2014 and then increased in 2017–2018, parallel to an increase in serotype 3, and returned to levels similar to the PCV7 period. IPD incidence due to non-PCV13 serotypes increased progressively during the PCV10/PCV13 period to 63% above the PCV7 period incidence level in 2018.

In persons >65 years of age, overall IPD incidence declined by 22% between the PCV7 period and 2014 and increased by 19% during 2015–2018, returning to a level close to the PCV7 period incidence (−7%; Table 4; Figure 1). IPD incidence due to PCV7 and PCV10 non-7 serotypes declined by 86%–91% between the PCV7 period and 2018. The incidence of PCV13 non-10 serotypes initially declined until 2014, and then increased during 2015–2018 (parallel to a 44% increase in serotype 3) to return to a level close to the PCV7 period. The non-PCV13 IPD incidence increased gradually to exceed the PCV7 period incidence by 84% in 2018 and increased in each site by 38%–155%.

### Changes in IPD Incidence According to PCV Program Characteristics

Overall, IPD incidence in children <5 years of age decreased between the PCV7 period and 2018 by 33% (95% CI 12%–50%) in the sites with universal PCV13, 54% (95% CI 44%–61%) in the sites in Spain, and 46% (95% CI 25%–61%) in sites using PCV10 (Appendix Figure 2, panel A). In persons >65 years of age, the change in overall IPD incidence ranged from –13% to +2% (Appendix Figure 3, panel A). PCV10 non-7 serotype incidence declined by 80%–98% in both age and vaccine policy groups. PCV13 non-10 serotype incidence initially declined in the universal PCV13 and Spain sites until 2014 and fluctuated in PCV10 sites. It increased in all age and vaccine groups from 2015 onwards. In children <5 years of age, this change resulted in a 63%–69% decline in the 2 groups of PCV13 sites and a 49% increase in the sites using PCV10 between the PCV7 period and 2018. In persons >65 years of age, this resulted in a 26%–30% decline in the 2 groups of PCV13 sites and a 78% increase in the sites using PCV10 between the PCV7 period and 2018. In the same period, serotype 3 increased in all PCV and age groups, in particular in sites using PCV10 (+51% in children <5 years of age and +66% in persons >65 years of age), except in children <5 years of age in universal PCV13 sites (–22%; Appendix Figure 2, panel A, and Figure 3, panel A). Non-PCV13 incidence also rose in each vaccine policy and age group.

**Figure 2:**
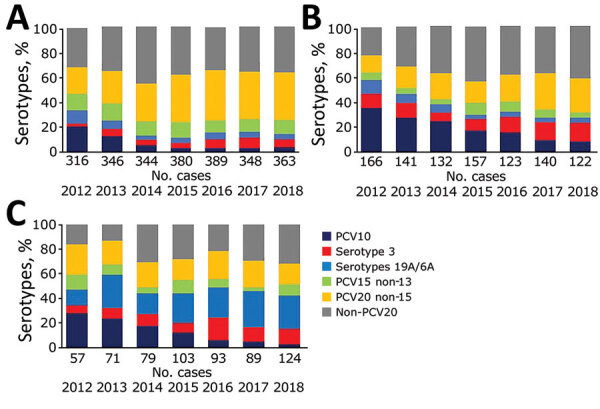
Percentage of cases of invasive pneumococcal disease caused by serotypes covered by PCV10, PCV13, PCV15, PCV20, and serotype 3 in children <5 years of age by vaccine policy, in 13 SpIDnet (*Streptococcus pneumoniae* Invasive Disease network) sites, Europe. A) Sites with universal PCV13 program; B) sites in Spain; C) sites using PCV10. Serotypes 19A/6A are included in PCV13 but not in PCV10 (in addition to serotype 3). PCV, pneumococcal conjugate vaccine.

**Figure 3:**
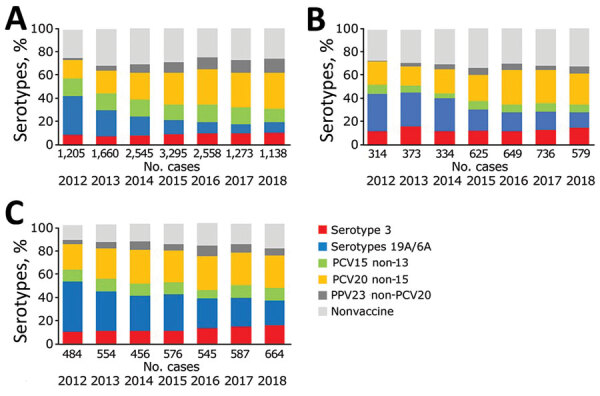
Percentage of cases of invasive pneumococcal disease caused by serotypes covered by PCV10, PCV13, PCV15, PCV20, and serotype 3 in persons >65 years of age by vaccine policy, in 13 SpIDnet (*Streptococcus pneumoniae* Invasive Disease network) sites, Europe. A) Sites with universal PCV13 program; B) sites in Spain; C) sites using PCV10. Serotypes 19A/6A are included in PCV13 but not in PCV10 (in addition to serotype 3). PCV, pneumococcal conjugate vaccine; PPV, pneumococcal polysaccharide vaccine.

Changes in overall IPD incidence were similar in sites with high or moderate uptake; incidence declined by 39%–50% in children (Appendix Figure 2, panel B) and no major change in IPD incidence occurred among persons >65 years of age (–12% to 8%; Appendix Figure 3, panel B). Changes in IPD incidence caused by vaccine serotype groups were similar across the levels of uptake, except for PCV7 serotypes, which decreased more in high-uptake sites than moderate-uptake sites and serotype 3 in children <5 years of age, which decreased in high-uptake sites and increased in moderate-uptake sites. Increases in non-PCV13 IPD incidence ranged from 81% to 85% in persons >65 years of age; in children, it increased more in high-uptake sites than moderate-uptake sites (223% vs. 56%; 95% CIs do not overlap).

Changes in overall IPD incidence were similar between sites with long and short duration of the PCV7 vaccination program (Appendix Figure 2, panel C, and Figure 3, panel C). Changes in vaccine serotype incidence also indicated similar patterns between the 2 groups, except for PCV13 non-10 serotype incidence, which decreased in long-duration sites and increased in short-duration sites. Serotype 3 incidence fluctuated in long-duration sites and rose by ≈70% in short-duration sites. In children, the increase in non-PCV13 serotype IPD was greater in short-duration sites than long-duration sites (214% vs. 93%; 95% CIs do not overlap).

### Heterogeneity Testing

In pooled analyses of the 13 sites, 𝜏^2^ was <0.5 in all analyses except for 2 in children <5 years of age and 1 in persons >65 years of age. In the analyses by vaccine policy group, 𝜏^2^ was <0.5 in 89% of analyses in children <5 years of age and 99% of analyses in persons >65 years of age, and 𝜏^2^ was >0.5 in the remaining 11% and 1% analyses (392 analyses per age group).

### Burden of Serotypes Covered by Current and Higher-Valent Vaccines

This analysis included 4,083 IPD cases in children <5 years of age and 21,250 cases in persons >65 years of age. The percentage of serotypes included in PCV13, PCV15, and PCV20 declined significantly (p<0.001) over the study period. In children <5 years of age, serotypes included in PCV13 represented 42% of 539 cases in 2012, those in PCV15 represented 53% of cases, and those in PCV20 represented 72% of cases. In 2018, for the same age group, PCV13 serotypes represented 23% of 609 IPD cases, PCV15 serotypes 32% of cases, and PCV20 serotypes 63% of cases. In persons ≥65 years of age, serotypes included in these vaccines accounted for 45% (PCV13), 57% (PCV15), and 75% (PCV20) of 2,003 cases in 2012 and 26% (PCV13), 36% (PCV15), and 65% (PCV20) of 2,481 cases in 2018. The percentage of IPD due to PCV20 nonPCV-13 serotypes increased from 30% to 41% in children <5 years of age and from 30% to 38% in persons >65 years of age (p<0.001). In 2018, the most frequent PCV20 non-13 serotypes were serotype 8 (7% of all IPD in children <5 years and 17% in persons >65 years), 10A and 12F in children <5 years (7% each) and 22F in persons >65 years (7%). The major contributor to the PCV20 non-13 increase was serotype 8 (3% of IPD cases in 2012 in children <5 years and 8% of IPD cases in 2012 in persons >65 years). The proportion of non-PCV20 serotype cases increased over time, from 28% in 2012 to 35% in 2018 in children <5 years and 27% in 2012 to 37% in 2018 in persons >65 years (p<0.002). The main non-PCV20 serotypes in 2018 were 23B (21% of non-PCV20), 24F (20%), and 15A (10%) in children <5 years and 9N (17%), 15A (9%), and 23A (8%) in persons >65 years.

Serotype 3 represented 9% of all cases and ranked as the most frequent serotype causing IPD in children <5 years; in persons >65 years, it represented 13% of all cases and ranked as the second most frequent serotype causing IPD. Excluding serotype 3 from the analysis, in 2018, PCV13 serotype represented 14% of IPD in children <5 years and 13% in persons >65 years, PCV15 represented 23% of IPD in both children <5 years and persons >65 years, and PCV20 serotype represented 54% of IPD in children <5 years and 51% of IPD in persons >65 years. PPV23 covered 73% of IPD cases in persons >65 years in 2018 or 60% if serotype 3 is excluded.

In 2018, proportions of serotypes included in PCV13, PCV15, and PCV20 were greater in sites using PCV10 than those using PCV13. In children <5 years, these percentages accounted for 43% (PCV13), 52% (PCV15) and 69% (PCV20) of 124 IPD cases in sites using PCV10 compared to 15% (PCV13), 26% (PCV15) and 63% (PCV20) of 363 IPD cases in PCV13 universal sites (Figure 2; p<0.001 except for PCV20, p = 0.18). In sites using PCV10, serotypes included in PCV15 and not in PCV10 amounted to 48% of cases; serotypes included in PCV20 and not PCV10 amounted to 65% of cases. In persons >65 years, PCV13 serotype proportions amounted to 36% of 664 cases in sites using PCV10 and 20% of 1,138 cases in PCV13 universal sites; PCV15 serotype proportions amounted to 46% of 664 cases in sites using PCV10 and 31% of 1,138 cases in PCV13 universal sites. PCV20 serotype proportions amounted to 73% of 664 cases in sites using PCV10 and 62% of 1,138 cases in PCV13 universal sites (Figure 3; p<0.001).

## Discussion

This study of 13 sites in Europe enabled us to estimate the overall effect of 8 years of the childhood PCV10 and PCV13 program on IPD in the age groups targeted directly and indirectly by vaccination. The results demonstrate an initial PCV effect on overall IPD incidence in all age groups during the first 4 years of the PCV10/PCV13 program; incidence declined by 22%–49% between the PCV7 period and 2014. This decline was followed, however, by an increase in incidence during 2015–2018, thereby reducing the benefits of PCV10/PCV13, especially among older adults. This reversing trend can be explained by a saturation of the PCV effect on vaccine serotype (PCV10/PCV13) incidence (i.e., no further decrease) alongside a gradual increase in non-PCV13 incidence and an increase in PCV13non10 incidence in more recent years.

The increase in non-PCV13 IPD incidence over time was observed in all analyses and ranged from 63% to 111% by age group between the PCV7 period and 2018. The greatest proportional increase in non-PCV13 incidence was observed in children, particularly in the sites with high PCV10/PCV13 uptake (+223% vs. +56% in sites with moderate uptake). This increase is likely due to a combination of vaccine-induced serotype replacement in carriage and disease (*25*–*27*) and secular trends in individual serotypes, but disentangling the 2 is difficult (*7*,*10*,*26*,*27*). Consequently, the overall effect of the current PCVs on overall IPD is diminishing in many EU settings, especially after 2014 (*6*,*8*,*25*,*28*).

In contrast to Europe and elsewhere, the Centers for Disease Control and Prevention Active Bacterial Core surveillance reported consistently stable rates of non-PCV13 serotype IPD in young children and older persons after childhood PCV13 introduction, even when vaccine uptake was very high (*8*,*11*). Several hypotheses have been proposed to explain this divergence (*8*,*12*,*29*). Of note, some similar serotypes are emerging in IPD in both regions (i.e., serotypes 8, 9N, and 15A), but their contribution to overall IPD differs (*8*).

A key finding of our study is that both PCV13 and PCV10 programs appear to result in a similar change of overall IPD incidence after 8 years of universal implementation (–33% for PCV13 and –46% for PCV10 in children and –13% for PCV13 to –3% for PCV10 in older adults), albeit with different serotype distribution of IPD cases. In particular, during the last years of the study (2015–2018), PCV13 non-10 IPD incidence increased in all vaccine policy and age groups because of an increase of serotype 3 in most groups, together with an increase in serotype 19A in PCV10 sites (data not shown). Serotype 3 remains a major cause of disease in both age groups, ranking first or second serotype in 2018, and its severity and associated high case-fatality rates, especially in older adults, raises concern (*30*,*31*). Genomic analyses in England and Wales suggested that a shift in clade distributions among invasive serotype isolates has led to the expansion of clade II since 2014, representing 50% of serotype 3 IPD isolates in 2018, which could account for the recent reemergence of this serotype (*32*). Increases in serotype 19A in PCV10 countries are also worrisome because this serotype is highly invasive and has been associated with high rates of antibiotic resistance (*33*,*34*).

In our study, the higher increase in non-PCV13 IPD incidence among children in the 8 sites with higher PCV uptake suggests a dose-response mechanism between uptake and nonvaccine rises. However, other factors, such as local differences in serotype dynamics, PCV used, vaccination schedule, timing, or uptake, might also play a role; of note, 3 of 4 sites with moderate uptake were in Spain.

Our study also provides evidence of a powerful and rapid indirect effect of the childhood PCV10/PCV13 vaccination in older adults, most of whom did not receive PCV13. Of note, changes in vaccine serotype IPD in older adults follow the same pattern of serotype changes in children with very limited delay but at a lower magnitude. As several other studies (*25*,*35*–*37*) have demonstrated, our study suggests that the maximum effect of PCV10/PCV13 has been reached; further declines in overall and vaccine-serotype IPD are unlikely to occur in countries with a high vaccine uptake and a mature PCV program.

The emergence of >2 new higher-valent PCVs is reassuring. In 2018, the serotypes in PCV15 covered around one third of IPD cases, and PCV20 covered around two thirds of IPD cases. Whether these 2 vaccines protect against serotype 3 IPD, however, remains to be established. Among the PCV20 non-13 serotypes, 4 serotypes ranked in the 5 top serotypes in children and older adults in 2018: serotype 8 in both age groups, 10A and 12F in children, and 22F in adults. Rises in serotype 8 have also been observed elsewhere (*1*,*25*,*30*,*35*,*38*). The proportion of non-PCV20 cases represented more than a third of all IPD cases in 2018 and have increased substantially since 2012. Of the top non-PCV20 serotypes in 2018 (23A, 23B, 24F, 15A, and 9N), only 9N is included in PPV23. Serotype 24F also ranked high in Italy and Germany during 2014–2016 (*5*,*26*). Including these serotypes in future vaccines might be beneficial, although higher-valency PCVs are likely to have only temporary benefit because of serotype replacement (*5*,*10*,*37*). Alternative strategies that provide serotype-independent protection, such as vaccines using antigens common to all serotypes, are desperately needed.

Our study’s first limitation is that methods comparing incidence before and after the start of a new vaccine program are prone to biases, whereby changes in other factors over time, such as surveillance methodologies, other health interventions, and secular trends of individual serotypes, might be attributed to the vaccination program (*18*). We partly addressed this limitation by adjusting for surveillance sensitivity and missing serotype data. Second, our network presents heterogeneity across sites because of differences in healthcare practices and vaccination policies (including duration of PCV7 use). We attempted to address this heterogeneity by using random effects meta-analysis and performing analysis stratified by PCV program characteristics. We assumed that these differences across sites would be constant over time, limiting their influence on relative measures of effect such as the IRR. Moreover, the τ^2^ values suggest limited statistical heterogeneity.

Our results suggest that the benefits of childhood PCV10/PCV13 programs on IPD incidence might level off after 8 years of vaccination. The gradual increase in nonvaccine serotype incidence countered the decrease in vaccine serotypes in recent years. Our results indicate that PCV15 serotypes could add 10% coverage of IPD cases, and PCV20 serotypes could add 30%–38% coverage of IPD cases compared with PCV13. On the basis of current evidence, however, protection at population level might be short-lived, and vaccines providing serotype-independent protection are urgently needed. Impact studies across multiple countries with different vaccines and schedules are critical for assessing long-term effects. In addition, studies to learn more about the mechanisms leading to the rise of some serotypes, such as 3, 19A, and 8, are required, especially incorporating genetic tools.

Appendix Additional information about serotype replacement after introduction of 10-valent and 13-valent pneumococcal conjugate vaccines in 10 countries, Europe

## References

[1] European Centre for Disease Prevention and Control. Invasive pneumococcal disease–annual epidemiological report for 2017. Stockholm: The Centre; 2019.

[2] Shiri T, Datta S, Madan J, Tsertsvadze A, Royle P, Keeling MJ, et al. Indirect effects of childhood pneumococcal conjugate vaccination on invasive pneumococcal disease: a systematic review and meta-analysis. Lancet Glob Health. 2017;5:e51–9. 10.1016/S2214-109X(16)30306-0 27955789

[3] Hanquet G, Krizova P, Valentiner-Branth P, Ladhani SN, Nuorti JP, Lepoutre A, et al.; SpIDnet/I-MOVE+ Pneumo Group. Effect of childhood pneumococcal conjugate vaccination on invasive disease in older adults of 10 European countries: implications for adult vaccination. Thorax. 2019;74:473–82. 10.1136/thoraxjnl-2018-211767 30355641 PMC6484683

[4] Savulescu C, Krizova P, Lepoutre A, Mereckiene J, Vestrheim DF, Ciruela P, et al.; SpIDnet group. Effect of high-valency pneumococcal conjugate vaccines on invasive pneumococcal disease in children in SpIDnet countries: an observational multicentre study. Lancet Respir Med. 2017;5:648–56. 10.1016/S2213-2600(17)30110-8 28359798

[5] Weinberger R, von Kries R, van der Linden M, Rieck T, Siedler A, Falkenhorst G. Invasive pneumococcal disease in children under 16 years of age: Incomplete rebound in incidence after the maximum effect of PCV13 in 2012/13 in Germany. Vaccine. 2018;36:572–7. 10.1016/j.vaccine.2017.11.085 29258705

[6] Ladhani SN, Collins S, Djennad A, Sheppard CL, Borrow R, Fry NK, et al. Rapid increase in non-vaccine serotypes causing invasive pneumococcal disease in England and Wales, 2000-17: a prospective national observational cohort study. Lancet Infect Dis. 2018;18:441–51. 10.1016/S1473-3099(18)30052-5 29395999

[7] Hausdorff WP, Hanage WP. Interim results of an ecological experiment - Conjugate vaccination against the pneumococcus and serotype replacement. [Erratum in: Hum Vaccin Immunother. 2016;12:2478–81]. Hum Vaccin Immunother. 2016;12:358–74. 10.1080/21645515.2015.1118593 26905681 PMC5049718

[8] Lewnard JA, Hanage WP. Making sense of differences in pneumococcal serotype replacement. Lancet Infect Dis. 2019;19:e213–20. 10.1016/S1473-3099(18)30660-1 30709666

[9] Feikin DR, Kagucia EW, Loo JD, Link-Gelles R, Puhan MA, Cherian T, et al.; Serotype Replacement Study Group. Serotype-specific changes in invasive pneumococcal disease after pneumococcal conjugate vaccine introduction: a pooled analysis of multiple surveillance sites. PLoS Med. 2013;10:e1001517. 10.1371/journal.pmed.1001517 24086113 PMC3782411

[10] Weinberger DM, Malley R, Lipsitch M. Serotype replacement in disease after pneumococcal vaccination. Lancet. 2011;378:1962–73. 10.1016/S0140-6736(10)62225-8 21492929 PMC3256741

[11] Ahmed SS, Pondo T, Xing W, McGee L, Farley M, Schaffner W, et al. Early impact of 13-valent pneumococcal conjugate vaccine use on invasive pneumococcal disease among adults with and without underlying medical conditions—United States. Clin Infect Dis. 2020;70:2484–92. 10.1093/cid/ciz739 31402387

[12] Miller E, Andrews NJ, Waight PA, Slack MP, George RC. Herd immunity and serotype replacement 4 years after seven-valent pneumococcal conjugate vaccination in England and Wales: an observational cohort study. Lancet Infect Dis. 2011;11:760–8. 10.1016/S1473-3099(11)70090-1 21621466

[13] Falkenhorst G, Remschmidt C, Harder T, Hummers-Pradier E, Wichmann O, Bogdan C. Effectiveness of the 23-valent pneumococcal polysaccharide vaccine (PPV23) against pneumococcal disease in the elderly: systematic review and meta-analysis. PLoS One. 2017;12:e0169368. 10.1371/journal.pone.0169368 28061505 PMC5218810

[14] Schiffner-Rohe J, Witt A, Hemmerling J, von Eiff C, Leverkus F-W. Efficacy of PPV23 in preventing pneumococcal pneumonia in adults at increased risk–a systematic review and meta-analysis. PLoS One. 2016;11:e0146338. 10.1371/journal.pone.0146338 26761816 PMC4711910

[15] Stacey HL, Rosen J, Peterson JT, Williams-Diaz A, Gakhar V, Sterling TM, et al. Safety and immunogenicity of 15-valent pneumococcal conjugate vaccine (PCV-15) compared to PCV-13 in healthy older adults. Hum Vaccin Immunother. 2019;15:530–9. 10.1080/21645515.2018.1532249 30648919 PMC6605726

[16] Thompson A, Lamberth E, Severs J, Scully I, Tarabar S, Ginis J, et al. Phase 1 trial of a 20-valent pneumococcal conjugate vaccine in healthy adults. Vaccine. 2019;37:6201–7. 10.1016/j.vaccine.2019.08.048 31495592

[17] European Centre for Disease Prevention and Control. Generic protocol on enhanced surveillance for invasive pneumococcal disease at the EU/EEA level. Stockholm: ECDC; 2018.

[18] Hanquet G, Valenciano M, Simondon F, Moren A. Vaccine effects and impact of vaccination programmes in post-licensure studies. Vaccine. 2013;31:5634–42. 10.1016/j.vaccine.2013.07.006 23856332

[19] European Commission. COMMISSION IMPLEMENTING DECISION of 8 August 2012 amending Decision 2002/253/EC laying down case definitions for reporting communicable diseases to the Community network under Decision No 2119/98/EC of the European Parliament and of the Council. 2012 [cited 2018 Sep 1]. https://eur-lex.europa.eu/legal-content/EN/TXT/?uri=CELEX%3A32012D0506

[20] Lepoutre A, Varon E, Georges S, Dorléans F, Janoir C, Gutmann L, et al.; Microbiologists of Epibac; ORP Networks. Impact of the pneumococcal conjugate vaccines on invasive pneumococcal disease in France, 2001-2012. Vaccine. 2015;33:359–66. 10.1016/j.vaccine.2014.11.011 25448105

[21] Stock NK, Maly M, Sebestova H, Orlikova H, Kozakova J, Krizova P. The Czech surveillance system for invasive pneumococcal disease, 2008–2013: a follow-up assessment and sensitivity estimation. PLoS One. 2015;10:e0131117. 10.1371/journal.pone.0131117 26125583 PMC4488342

[22] Riley RD, Higgins JPT, Deeks JJ. Interpretation of random effects meta-analyses. BMJ. 2011;342(feb10 2):d549. 10.1136/bmj.d549 21310794

[23] Rücker G, Schwarzer G, Carpenter JR, Schumacher M. Undue reliance on I(2) in assessing heterogeneity may mislead. BMC Med Res Methodol. 2008;8:79. 10.1186/1471-2288-8-79 19036172 PMC2648991

[24] Andrews N, Kent A, Amin-Chowdhury Z, Sheppard C, Fry N, Ramsay M, et al. Effectiveness of the seven-valent and thirteen-valent pneumococcal conjugate vaccines in England: The indirect cohort design, 2006-2018. Vaccine. 2019;37:4491–8. 10.1016/j.vaccine.2019.06.071 31272872

[25] van der Linden M, Imöhl M, Perniciaro S. Limited indirect effects of an infant pneumococcal vaccination program in an aging population. [Erratum in: PLoS One. 2020;15:e0228126]. PLoS One. 2019;14:e0220453. 10.1371/journal.pone.0220453 31369597 PMC6675109

[26] Camilli R, D’Ambrosio F, Del Grosso M, Pimentel de Araujo F, Caporali MG, Del Manso M, et al.; Pneumococcal Surveillance Group. Impact of pneumococcal conjugate vaccine (PCV7 and PCV13) on pneumococcal invasive diseases in Italian children and insight into evolution of pneumococcal population structure. Vaccine. 2017;35(35 Pt B):4587–93. 10.1016/j.vaccine.2017.07.010 28716556

[27] De Wals P, Lefebvre B, Deceuninck G, Longtin J. Incidence of invasive pneumococcal disease before and during an era of use of three different pneumococcal conjugate vaccines in Quebec. Vaccine. 2018;36:421–6. 10.1016/j.vaccine.2017.11.054 29224962

[28] Naucler P, Galanis I, Morfeldt E, Darenberg J, Örtqvist Å, Henriques-Normark B. Comparison of the impact of pneumococcal conjugate vaccine 10 or pneumococcal conjugate vaccine 13 on invasive pneumococcal disease in equivalent populations. Clin Infect Dis. 2017;65:1780–9. 10.1093/cid/cix685 29020171 PMC5848315

[29] Hausdorff WP. Pneumococcal conjugate vaccines in different settings. Lancet Infect Dis. 2019;19:1283–4. 10.1016/S1473-3099(19)30623-1 31782390

[30] Amin-Chowdhury Z, Collins S, Sheppard C, Litt D, Fry NK, Andrews N, et al. Characteristics of invasive pneumococcal disease (IPD) caused by emerging serotypes after the introduction of the 13-valent pneumococcal conjugate vaccine (PCV13) in England; prospective observational cohort study, 2014–18. Clin Infect Dis. 2020;71:e235–43. 10.1093/cid/ciaa043 31955196

[31] Ciruela P, Broner S, Izquierdo C, Pallarés R, Muñoz-Almagro C, Hernández S, et al.; Catalan Working Group on Invasive Pneumococcal Disease. Indirect effects of paediatric conjugate vaccines on invasive pneumococcal disease in older adults. [Erratum in: Int J Infect Dis. 2020;91:206]. Int J Infect Dis. 2019;86:122–30. 10.1016/j.ijid.2019.06.030 31283992

[32] Groves N, Sheppard CL, Litt D, Rose S, Silva A, Njoku N, et al. Evolution of *Streptococcus pneumoniae* serotype 3 in England and Wales: a major vaccine evader. Genes (Basel). 2019;10:845. 10.3390/genes10110845 31731573 PMC6896183

[33] Mališová L, Urbášková P, Jakubů V, Španělová P, Kozáková J, Musílek M, et al. Surveillance of antibiotic resistance of Streptococcus pneumoniae in the Czech Republic, respiratory study results, 2010-2017. Epidemiol Mikrobiol Imunol. 2019;68:75–81. 31398980

[34] Del Amo E, Esteva C, Hernandez-Bou S, Galles C, Navarro M, Sauca G, et al.; Catalan Study Group of Invasive Pneumococcal Disease. Serotypes and clonal diversity of Streptococcus pneumoniae causing invasive disease in the era of PCV13 in Catalonia, Spain. PLoS One. 2016;11:e0151125. 10.1371/journal.pone.0151125 26953887 PMC4783110

[35] Tin Tin Htar M, Morato Martínez J, Theilacker C, Schmitt H-J, Swerdlow D. Serotype evolution in Western Europe: perspectives on invasive pneumococcal diseases (IPD). Expert Rev Vaccines. 2019;18:1145–55. 10.1080/14760584.2019.1688149 31682762

[36] Corcoran M, Vickers I, Mereckiene J, Murchan S, Cotter S, Fitzgerald M, et al. The epidemiology of invasive pneumococcal disease in older adults in the post-PCV era. Has there been a herd effect? Epidemiol Infect. 2017;145:2390–9. 10.1017/S0950268817001194 28712384 PMC9148822

[37] Izurieta P, Bahety P, Adegbola R, Clarke C, Hoet B. Public health impact of pneumococcal conjugate vaccine infant immunization programs: assessment of invasive pneumococcal disease burden and serotype distribution. Expert Rev Vaccines. 2018;17:479–93. 10.1080/14760584.2018.1413354 29241390

[38] González-Díaz A, Càmara J, Ercibengoa M, Cercenado E, Larrosa N, Quesada MD, et al. Emerging non-13-valent pneumococcal conjugate vaccine (PCV13) serotypes causing adult invasive pneumococcal disease in the late-PCV13 period in Spain. Clin Microbiol Infect. 2020;26:753–9. 10.1016/j.cmi.2019.10.034 31756452

