# Supplementary material for: Serotype Replacement after Introduction of 10-Valent and 13-Valent Pneumococcal Conjugate Vaccines in 10 Countries, Europe
Source: Emerg Infect Dis. 2022 Jan;28(1):127–38. doi: 10.3201/eid2801.210734 (PMC8714201; doi:10.3201/eid2801.210734)
Supplement: Appendix — Additional information about serotype replacement after introduction of 10-valent and 13-valent pneumococcal conjugate vaccines in 10 countries, Europe [file 21-0734-Techapp-s1.pdf]

Article DOI: https://doi.org/10.3201/eid2801.210734

Serotype Replacement after Introduction of 10-Valent and 13-Valent Pneumococcal Conjugate Vaccines in 10 Countries, Europe

Appendix

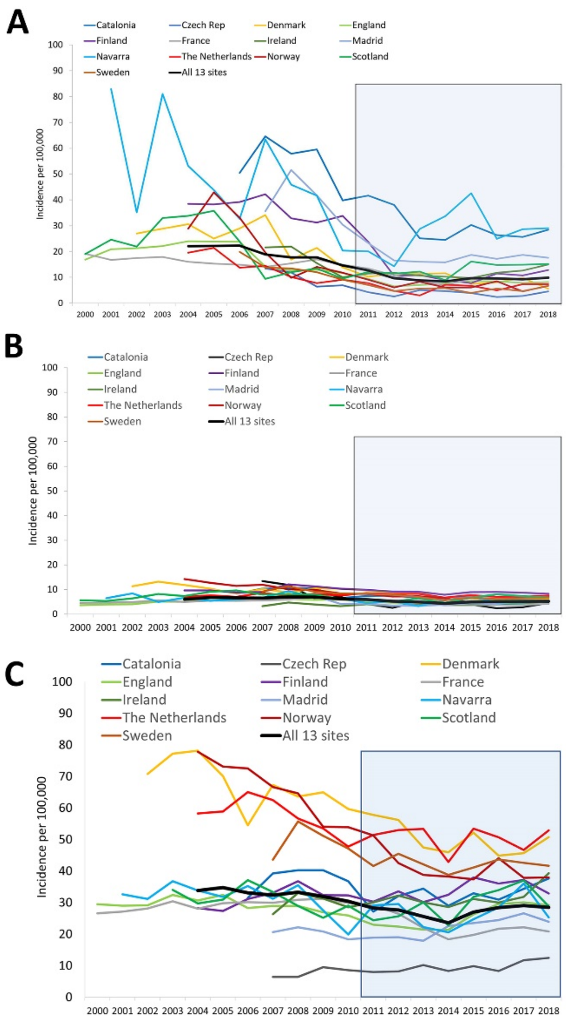

Appendix Figure 1. Overall incidence rates of invasive pneumococcal disease, pooled and per site, by year (2000–2018), 13 SpIDnet sites, Europe. A) Incidence rates in children <5 years of age, B) in persons 5–64 years of age, C) in persons >65 years of age. Light blue square indicates period of PCV10/PCV13 program.

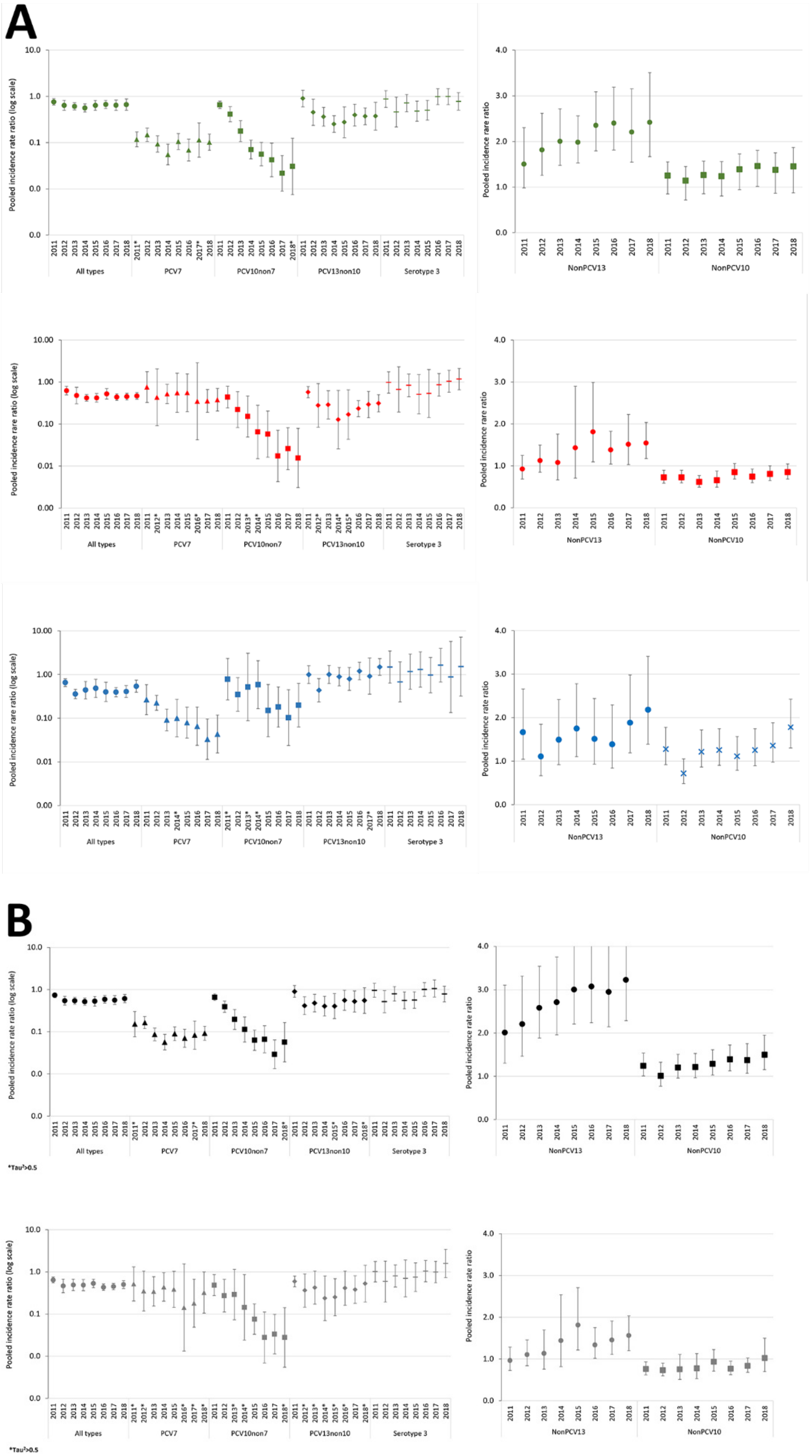
A
Pooled incidence rate ratio (log scale)
Pooled incidence rare ratio
All types
PCV7
PCV10non7
PCV13non10
Serotype 3
NonPCV13
NonPCV10
B
Pooled incidence rate ratio
*Tau²>0.5

C

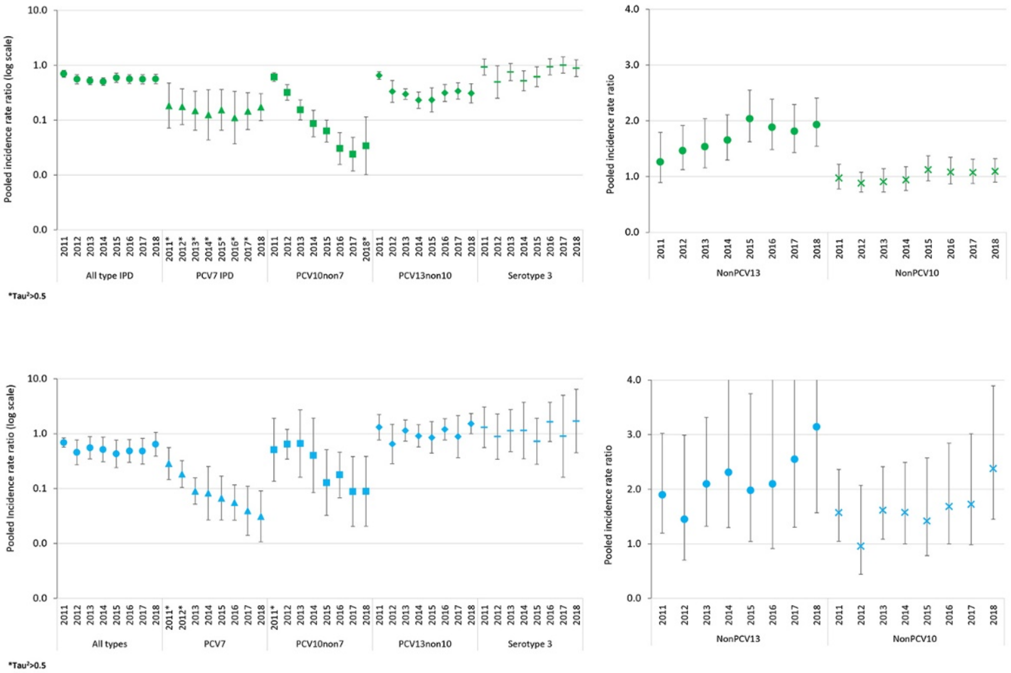

Appendix Figure 2. Pooled incidence rate ratio (IRR) of invasive pneumococcal disease in children <5 years of age per year of PCV10/PCV13 year program (2011–2018) compared with the PCV7 period in 13 SpIDnet sites, Europe. A) Pooled IRR by vaccine policy (top: universal PCV13 vaccination, 6 sites; middle: 3 sites in Spain; bottom: universal vaccination with PCV10 with or without PCV13, 4 sites). B) Pooled IRR by level of uptake (top: high uptake, 9 sites; bottom: moderate uptake, 4 sites). C) Pooled IRR by duration of PCV7 vaccination (top: $\geq$3 years PCV7, 9 sites; bottom: <3 years PCV7, 4 sites). PCV, pneumococcal conjugate vaccine.

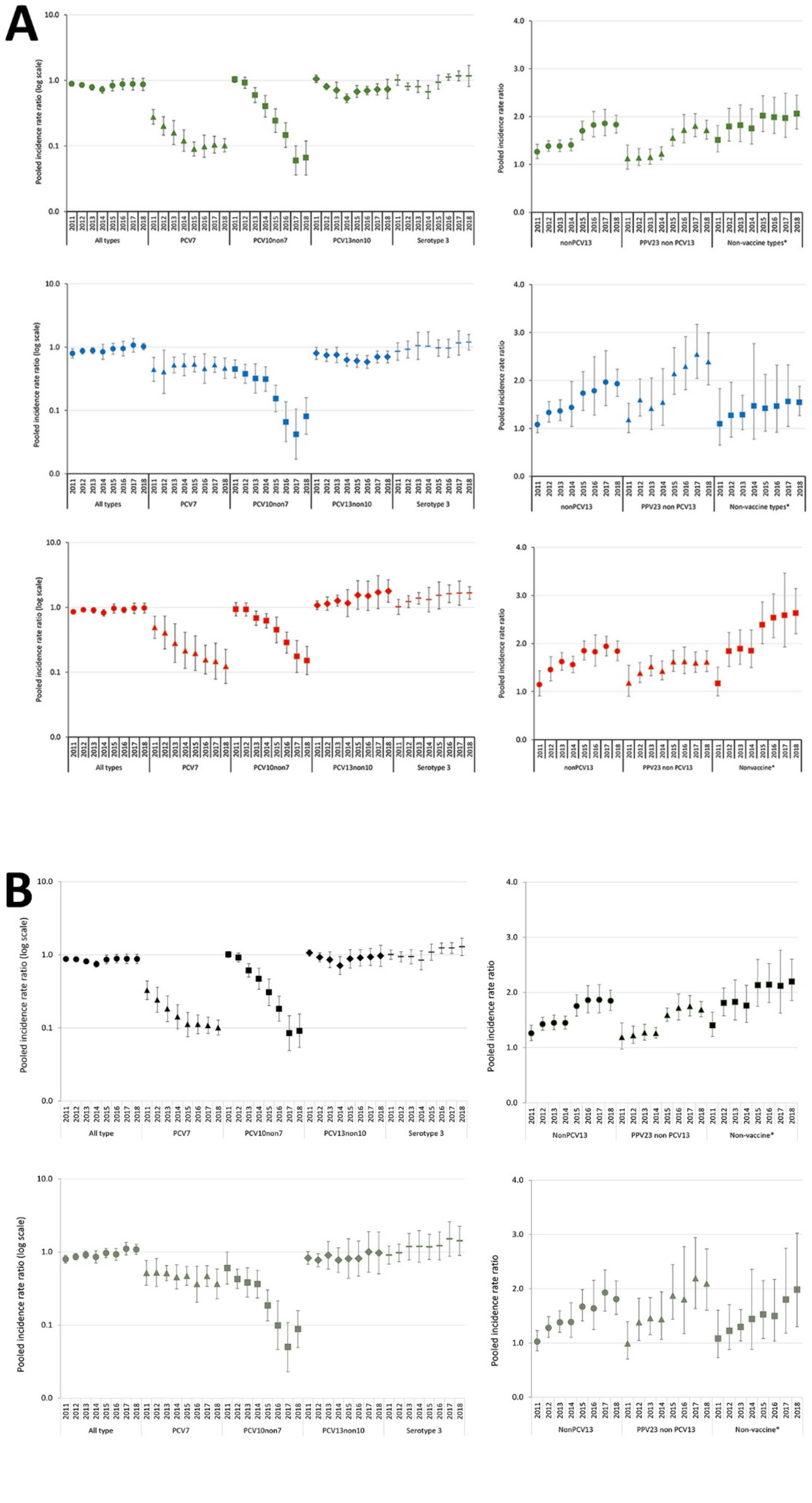
A
B
Pooled incidence rate ratio (log scale)
Pooled incidence rate ratio
All types
PCV7
PCV10non7
PCV13non10
Serotype 3
nonPCV13
PPV23 non PCV13
Non-vaccine types*
Nonvaccine*
All type
NonPCV13
Non-vaccine*

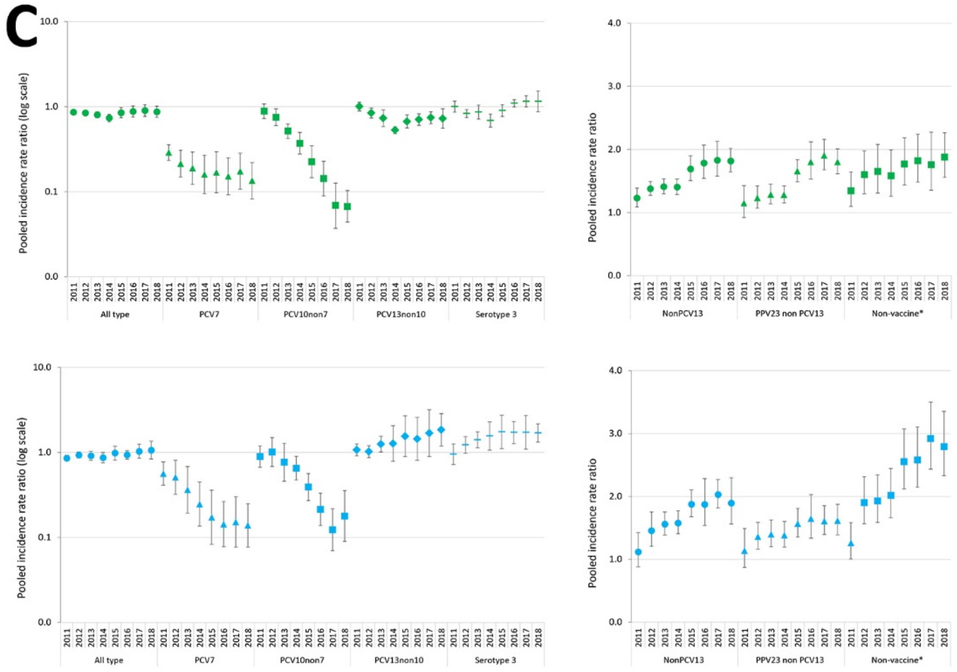

Appendix Figure 3. Pooled incidence rate ratio (IRR) of invasive pneumococcal disease in adults >65 years of age per year of PCV10/PCV13 program (2011–2018) compared with the PCV7 period. A) Pooled IRR by vaccine policy (top: universal PCV13 vaccination, 6 sites; middle: 3 sites in Spain; bottom: universal vaccination with PCV10 with or without PCV13, 4 sites). B) Pooled IRR by level of uptake (top: high uptake, 9 sites; bottom: moderate uptake, 4 sites). C) Pooled IRR by duration of PCV7 vaccination (top: >3 years PCV7, 9 sites; bottom: <3 years PCV7, 4 sites). *Serotypes not in PPV23 and not in PCV13. PCV, pneumococcal conjugate vaccine; PPV, pneumococcal polysaccharide vaccine.
